# PLS-Prediction and Confirmation of Hydrojuglone Glucoside as the Antitrypanosomal Constituent of *Juglans* Spp.

**DOI:** 10.3390/molecules200610082

**Published:** 2015-05-29

**Authors:** Therese Ellendorff, Reto Brun, Marcel Kaiser, Jandirk Sendker, Thomas J. Schmidt

**Affiliations:** 1 Institute of Pharmaceutical Biology and Phytochemistry, University of Münster, PharmaCampus, Corrensstr. 48, Münster D-48149, Germany; E-Mails: therese.ellendorff@uni-muenster.de (T.E.); jandirk.sendker@uni-muenster.de (J.S.); 2 Swiss Tropical and Public Health Institute (Swiss TPH), Socinstraße 57, Basel CH-4002, Switzerland; E-Mails: reto.brun@unibas.ch (R.B.); marcel.kaiser@unibas.ch (M.K.); 3 University of Basel, Petersplatz 1, Basel CH-4003, Switzerland

**Keywords:** *Juglans*, Juglandaceae, *Trypanosoma brucei rhodesiense*, naphthoquinones, hydrojuglone glucoside, LC-MS, Partial Least Squares

## Abstract

Naphthoquinones (NQs) occur naturally in a large variety of plants. Several NQs are highly active against protozoans, amongst them the causative pathogens of neglected tropical diseases such as human African trypanosomiasis (sleeping sickness), Chagas disease and leishmaniasis. Prominent NQ-producing plants can be found among *Juglans* spp. (Juglandaceae) with juglone derivatives as known constituents. In this study, 36 highly variable extracts were prepared from different plant parts of *J. regia, J. cinerea* and *J. nigra*. For all extracts, antiprotozoal activity was determined against the protozoans *Trypanosoma cruzi*, *T. brucei rhodesiense* and *Leishmania donovani*. In addition, an LC-MS fingerprint was recorded for each extract. With each extract’s fingerprint and the data on *in vitro* growth inhibitory activity against *T. brucei rhodesiense* a Partial Least Squares (PLS) regression model was calculated in order to obtain an indication of compounds responsible for the differences in bioactivity between the 36 extracts. By means of PLS, hydrojuglone glucoside was predicted as an active compound against *T. brucei* and consequently isolated and tested *in vitro*. In fact, the pure compound showed activity against *T. brucei* at a significantly lower cytotoxicity towards mammalian cells than established antiprotozoal NQs such as lapachol.

## 1. Introduction

Protozoal infections are a major cause of suffering and death in tropical regions and especially in developing countries. The WHO lists human African trypanosomiasis (HAT or sleeping sickness), Chagas disease and different leishmaniases including visceral leishmaniasis (Kala-Azar), as neglected tropical diseases with protozoan parasites as causative pathogens (the hemoflagellates *Trypanosoma brucei rhodesiense*, *T. cruzi* and *Leishmania donovani*, respectively) [1,2].

Naphthoquinones (NQs) constitute a class of secondary plant metabolites with frequently reported activity against these pathogens. NQs are immanently susceptible to oxidative reactions as well as to the addition of electrophiles via Michael-addition, and products of such reactions have been described as plant constituents. This includes for example dimeric NQs like diospyrin, which result from oxidative coupling of monomers and may also exhibit antiprotozoal activities [3,4,5,6]. NQs and other quinones may disturb the cellular redox status and thereby provoke the generation of reactive oxygen species (ROS) by redox cycling. Because their redox metabolism significantly differs from that of other eukaryotic cells, protozoans of the family *Trypanosomatidae* are strongly impaired by ROS. Yet, their poor bioavailability and toxicity to mammalian cells has limited the application of NQs and the necessity to find related structures with improved activity and/or reduced side effects has been stated [5,6,7].

ROS mainly arising from the photosynthetic apparatus of herbal materials under conditions of stress or senescence have been shown to significantly impact on secondary metabolites [8,9,10]. Due to their susceptibility to chemical modifications by e.g., oxidative coupling we hypothesized that the oxidative environment provided in drying or senescing leaves of NQ-bearing plants should yield new NQ derivatives with possibly improved pharmacological properties.

We decided for a chemometric approach using Partial Least Squares Regression (PLS) in order to readily detect such compounds in extract of *Juglans* spp. (Juglandaceae)—a genus that generates the NQ juglone from its precursor hydrojuglone [11]. PLS has first been introduced to phytochemistry as a predictive model for the anti-plasmodial activity of *Artemisia annua* L., Asteraceae [12] and was later used as a reflective model allowing for the identification of analytical signals correlated with bioactivity [13,14,15]. Our study aims at reflecting on PLS models in order to identify compounds from *Juglans* spp. that show antiprotozoal activity by correlating multiple samples’ bioactivities with their LC-MS fingerprints.

## 2. Results and Discussion

Due to their high content of NQ-related compounds and abundant local availability, *Juglans* spp. appeared an interesting group of plants to investigate for antiprotozoal activity. To this end, samples of different plant parts (leaves, pericarps, bark, male flowers) in varying developmental stages, including senescent parts, were collected from *Juglans regia* L. (JR), *Juglans cinerea* L. (JC) and *Juglans nigra* L. (JN) (all Juglandaceae) and either freeze- or air-dried. Extracts were prepared with solvents of different polarity and extraction methods (see Experimental) yielding a total of 36 different samples. This procedure was chosen in order to maximize the chemical variability in the examined samples which is a prerequisite for successful application of multivariate data analysis methods. All extracts were tested for *in vitro* growth-inhibitory activity against the *Trypanosomatidae Trypanosoma brucei rhodesiense* Plimmer & Bradford (TB), *T. cruzi* Chagas (TC) and *Leishmania donovani* (Laveran et Mesnil) Ross (LD). Each sample was tested against each parasite at two different concentrations, *i.e.*, 2 and 10 μg/mL. The results, along with the main characteristics of each extract, are listed in Table 1.

**Table 1:** Bioactivities of different extracts from *Juglans regia* (JR), *Juglans cinerea* (JC1 and JC2) and *Juglans nigra* (JN) against *Trypanosoma brucei rhodesiense* (TB), *Trypanosoma cruzi* (TC) and *Leishmania donovani* (LD). FD = freeze dried, AD = air dried. Refer to Experimental section for extraction procedures A–G.

No.	Species	Plant Part	State	Drying	Extraction	% Inhibition at 2 μg/mL Against			% Inhibition at 10 μg/mL Against		
						TB	TC	LD	TB	TC	LD
1	JR	Male flower	Green	FD	D	83.0	6.8	21.5	100.0	0.9	29.9
2	JR	Pericarp	Green	FD	E	4.7	7.9	16.7	36.7	11.7	21.1
3	JR	Bark	n.a.	FD	D	99.5	5.9	16.6	99.1	12.5	54.4
4	JC2	Leaf	Green	FD	D	68.9	0.0	0.0	100.0	7.7	0.0
5	JC1	Pericarp	Green	FD	D	23.2	2.6	24.6	100.0	0.9	23.3
6	JR	Leaf	Green	FD	F	15.9	0.0	24.9	78.4	0.0	23.6
7	JN	Leaf	Green	FD	D	45.2	0.4	26.4	100.0	5.7	24.2
8	JR	Pericarp	Green	FD	G	20.0	5.3	25.5	100.0	9.3	27.4
9	JN	Leaf	Green	FD	F	85.6	0.0	28.3	100.0	10.3	40.8
10	JR	Pericarp	Green	FD	D	24.1	0.0	24.1	100.0	0.0	28.7
11	JR	Male flower	Senescent	FD	D	9.7	11.1	23.5	18.2	5.5	20.3
12	JN	Pericarp	Senescent	None	A	5.9	0.0	19.1	43.0	23.8	18.4
13	JC2	Pericarp	Green	FD	D	15.5	0.0	16.3	99.8	13.3	23.2
14	JR	Pericarp	Green	FD	D	26.1	4.4	16.6	100.0	23.5	24.5
15	JC1	Leaf	Senescent	FD	D	95.6	0.2	2.6	100.0	3.0	0.0
16	JN	Pericarp	Green	None	A	15.1	0.0	23.9	76.5	15.7	14.3
17	JN	Pericarp	Senescent	None	B	12.3	5.1	22.5	39.4	10.1	19.3
18	JR	Leaf	Senescent	FD	C	9.5	11.8	25.5	22.7	10.5	27.6
19	JR	Leaf	Green	FD	C	73.4	0.0	26.9	100.0	8.3	57.9
20	JN	Pericarp	Green	None	B	42.7	0.0	24.7	100.0	12.9	23.5
21	JR	Leaf	Senescent	FD	B	30.8	0.0	24.5	100.0	11.9	27.7
22	JR	Leaf	Green	FD	D	51.8	3.1	21.1	100.0	1.7	27.7
23	JR	Leaf	Green	FD	D	17.2	9.1	18.0	98.7	4.0	19.8
24	JC1	Leaf	Green	FD	D	60.7	6.8	15.6	100.0	0.0	23.9
25	JR	Leaf	Senescent	AD	D	2.5	8.5	16.4	24.6	18.7	25.2
26	JR	Pericarp	Green	FD	B	43.4	5.6	12.1	100.0	4.4	50.8
27	JR	Leaf	Green	AD	D	13.9	6.9	26.5	22.8	4.7	22.1
28	JN	Leaf	Senescent	FD	D	76.3	8.1	26.2	100.0	0.0	20.1
29	JC2	Leaf	Senescent	FD	D	15.3	13.0	25.9	80.4	15.2	18.6
30	JR	Leaf	Green	FD	B	15.2	6.7	27.6	94.3	15.4	18.2
31	JN	Pericarp	Green	FD	D	54.4	9.7	22.7	100.0	0.0	29.0
32	JN	Leaf	Green	FD	C	8.7	12.1	24.3	19.9	11.7	38.7
33	JC1	Leaf	Green	FD	C	5.7	8.4	20.8	8.8	0.0	34.9
34	JR	Pericarp	Green	FD	C	69.4	8.7	29.2	100.0	18.1	100.0
35	JN	Pericarp	Green	FD	C	5.7	4.8	17.3	71.0	6.0	27.8
36	JN	Pericarp	Green	AD	C	100.0	0.0	20.7	100.0	16.1	69.3

Activities against TC and LD were quite low and hardly ever exceeded 20% or 50% inhibition, respectively. In contrast, multiple extracts from the sample collection frequently reached inhibition rates >90% against TB, while several other extracts showed poor or no observable activity under the test conditions. In order to test our hypothesis that senescent material should contain new active NQs, we compared by paired *t*-test the activities of those extracts of the same kind whose original materials only differed in their physiological state (e.g., No. 7 with No. 28). Senescent samples showed a significantly lower activity against TB than green samples (*p*-value 0.04). Consequently, this hypothesis is rejected.

In order to identify chemical constituents in the plant materials which are responsible for the variability in the biological response data (*i.e.*, compounds accounting for high activity of certain samples in comparison with others), we chose to apply a method of multivariate statistics, namely, Partial Least Squares (PLS) regression, correlating biological activity with LC-MS analytical data. As for any correlative analysis, a meaningful PLS regression model should benefit from an adequate number of samples sufficiently varying within both the independent and dependent variables, *i.e.*, in this case the chemical profile and bioactivity, respectively, which was assumed to be the case in the present set of anti-TB data.

**Figure 1:**
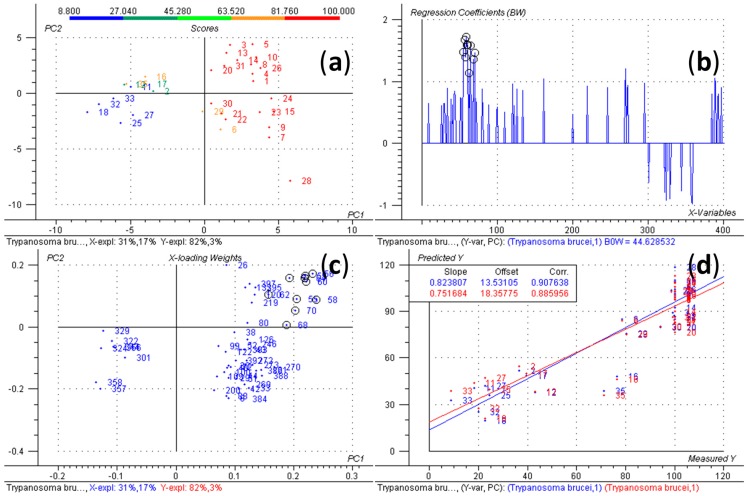
PLS model with activity data against *Trypanosoma brucei rhodesiense*. Numbers in scores plot (**a**) and response plot (**d**) relate to samples numbers of Table 1. Colour code in (**a**) indicates the samples’ inhibitory activity from the lowest (8.8%, blue) to the highest value (100%, red). Red colour in (**d**) indicates cross-validated data. Numbers in regression coefficient plot (**b**) and loadings plot (**c**) relate to bucket numbers. Buckets representing (**1**) are highlighted by circles.

To this end, each extract’s chemical fingerprint was recorded by HPLC/ESI-QTOF-MS and processed into a data matrix suitable for PLS using Bruker Daltonics ProfileAnalysis 2.0 (Bruker Daltonik GmbH, Bremen, Germany, 2010). In particular, processing involved a bucketing procedure based on the detection of isotope patterns appearing as chromatographic peaks (program function “Find Molecular Features”). This matrix of independent variables, consisting of 401 variables, each representing an *m*/*z*-value: Retention time pair, was then exported to Camo Process AS the Unscrambler v. 9.2 (Camo Process AS, Oslo, Norway, 2005), subjected to unit variance scaling and amended by adding the bioactivity data as dependent variables before constructing a PLS model. The resulting PLS-model for activity against TB is depicted in Figure 1. High values for explained variance R^2^ and variance predicted by leave-one-out cross validation Q^2^ indicate that a well-fitting regression model was obtained (R^2^ = 0.908/Q^2^ = 0.886). Reflection on this model revealed that the buckets No. 68 (11.0 min: *m*/*z* 177.06) and 70 (11.0 min: *m*/*z* 339.12) along with some buckets of minor intensity (Table 2) show strong positive loading weights on PC1. Consequently, these buckets can be considered as important predictors for bioactivity. In fact, all buckets represent signals of pseudomolecular ions or fragments in the mass spectrum of the same compound which is thus very likely to represent a compound with antitrypanosomal activity.

**Table 2:** Signals of the mass spectrum of (**1**) (sample No. 28) and related buckets as identified by PLS. All fragment ions were confirmed by MS/MS of *m*/*z* 339 [M + H]^+^.

Mass Spectrum of (1)		Bucket Table		
*m*/*z* (Rel. Int.)	Ion	No.	Name	Loading Weight on PC1
699.1985 (7)	[2M + Na]^+^	54	10.8 min: 699.19 *m*/*z*	n.a.
677.2157 (10)	[2M + H]^+^	67	10.9 min: 677.21 *m*/*z*	n.a.
501.1665 (11)	[2M − C_10_H_8_O_3_ + H]^+^	64	10.9 min: 501.18 *m*/*z*	0.218
377.0669 (2)	[M + K]^+^	62	10.9 min: 377.10 *m*/*z*	0.157
361.0930 (9)	[M + Na]^+^	61	10.9 min: 361.10 *m*/*z*	n.a.
356.1378 (3)	[M + NH_4_]^+^	60	10.9 min: 356.15 *m*/*z*	0.221
339.1117 (43)	[M + H]^+^	70	11.0 min: 339.12 *m*/*z*	0.202
321.1000 (4)	fragments of [M + H]^+^	59	10.9 min: 321.11 *m*/*z*	0.220
303.0907 (7)		58	10.9 min: 303.10 *m*/*z*	0.238
285.0785 (2)		57	10.9 min: 285.09 *m*/*z*	0.192
243.0685 (3)		56	10.9 min: 243.08 *m*/*z*	0.231
201.0576 (3)		55	10.9 min: 201.06 *m/z*	0.205
177.0570 (100)		68	11.0 min: 177.06 *m*/*z*	0.187

n.a.: Not available—buckets were excluded by Martens’ Uncertainty Test.

**Figure 2:**
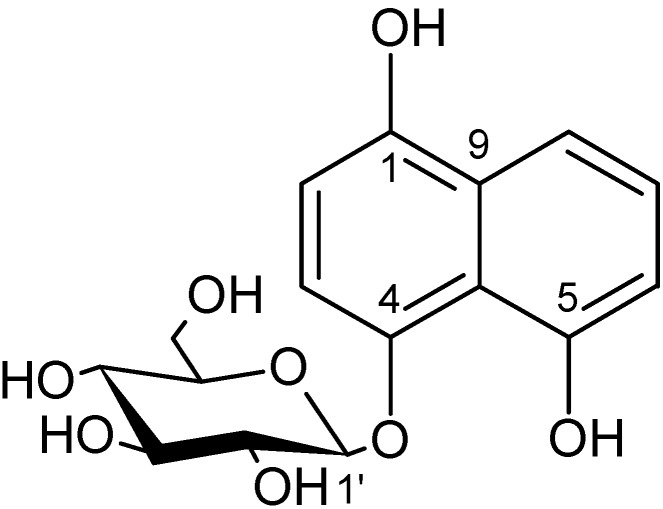
Structure of hydrojuglone glucoside (**1**).

This compound **1** was isolated in three steps (see Experimental Section) from a methanol extract of green JR-leaves, which was the most abundant source material. The molecular formula was established as C_16_H_18_O_8_ from the exact *m*/*z* and isotope pattern of the [M + H]^+^; fragmentation indicates loss of a hexose moiety (NFL of 162.0547 amu) to yield the aglycone fragment at *m*/*z* 177.0570 (C_10_H_8_O_3_). Further, all fragments indicated by PLS were present in the MS² spectrum and could be explained by Metfrag [16]. The hexose was identified as β-glucose by the coupling constant of its anomeric proton (^3^*J*_1,2_ = 7.6 Hz) and its ^13^C-NMR shift values [17]. The remaining ^1^H-NMR data indicate five aromatic protons for the aglycone, allocated to an AMX and an AX spin system, each one showing at least one coupling constant between 6.7 and 8.4 Hz. HSQC and ^13^C-NMR data are consistent with five aromatic methine carbons and two quaternary carbons resonating between 108.21 and 128.86 ppm as well as three hydroxylated quaternary carbons with signals around 150 ppm. The HMBC spectrum showed a cross signal between the anomeric proton and a hydroxylated aromatic carbon in vicinity to the AX spin system and without an aromatic proton in peri-position. Thus, the aglycone was assigned as hydrojuglone and the entire molecule **1** as hydrojuglone β-glucoside (Figure 2). All spectroscopic data are in agreement with published values (NMR: [18]; UV and MS: [19]).

Pure **1** was consequently tested in the cellular assays and found to be active against TB (IC_50_ = 6.1 μM, SI = 20.0) and LD (IC_50_ = 16.7 μM, SI = 7.3). The cytotoxicity of **1** against mammalian cells was low (L6 rat skeletal myoblasts; IC_50_ = 122.4 μM). In comparison to the activity data of selected NQ and the chemically related benzohydroquinone glucoside arbutin which were also tested (Table 3), **1** shows the best combination of antitrypanosomal activity and toxicity. Even though its IC_50_ value against TB is considerably higher than those of the quinones juglone, 1,4-naphothoquinone, plumbagin and shikonin, its low cytotoxicity with a selectivity index of about 20 makes it the most interesting compound in this series.

**Table 3:** IC_50_ values against *Trypanosoma brucei rhodesiense* (TB), *T. cruzi* (TC), *Leishmania donovani* (LD) and rat skeletal myoblasts (L6). Selectivity indices (SI) represent the ratio of cytotoxic over antiparasitic IC_50_ values.

Compound	IC_50_ (μM)				SI		
	TB	TC	LD	L6	TB	TC	LD
Hydrojuglone glucoside (1)	6.12	169.40	16.65	122.48	20.0	0.7	7.4
Juglone	1.62	>100	2.02	21.03	13.0	<0.1	10.4
1,4-Naphthoquinone	0.58	9.24	2.38	6.39	11.1	0.7	2.7
Lawson	101.15	37.36	1.99	20.63	0.2	0.6	10.3
2,2′-bis-(3-Hydroxy-1,4-naphthoquinone)	68.79	236.99	7.34	61.56	0.9	0.3	8.4
Plumbagin	0.49	3.59	0.88	2.87	5.9	0.8	3.3
Lapachol	16.40	17.56	3.33	26.57	1.6	1.5	8.0
Shikonin	0.03	0.27	0.12	0.06	2.1	0.2	0.5
Arbutin (1,4-benzoquinone glucoside)	236.40	244.10	>367	>367	>1.5	>1.5	n.a
Positive controls	0.005	2.33	0.18	0.014			

n.a.: Not accessible (IC_50_ not reached at highest tested concentration); Positive controls: TB: Melarsoprol; TC: Benznidazole; LD: Miltefosine; L6: Podophyllotoxin.

## 3. Experimental Section

### 3.1. General Experimental Procedures

D_2_O was purchased from Merck Chemicals (Merck KGaA, Darmstadt, Germany). Chromatographic eluents and their additives were purchased from VWR International (VWR International GmbH, Darmstadt, Germany). Any other solvents or chemicals were purchased from Fisher Chemicals (Fisher Scientific, Schwerte, Germany).

### 3.2. Plant Material

Plant material of *Juglans regia* L. (JR), *J. cinerea* L. (JC) and *J. nigra* L. (JN) was collected in Münster, Germany, during the years 2011 and 2012. Plants were identified by T.E. From JC, two different individuals showing distinct chemical fingerprints were chosen (JC1 and JC2). For each species a voucher specimen was stored at IPBP, University of Münster (voucher numbers: JR: IPBP342; JC: IPBP340: JN: IPBP341). Different plant parts in different developmental states were used in order to achieve a high degree of diversity of the resulting extracts (see Table 1). Senescent leaves, flowers or fruits were collected in October and were characterized by chlorosis and significantly lowered detachment force. Plant material was either air-dried at ambient temperature, or freeze-dried after being ground in liquid nitrogen.

### 3.3. Extract Preparation

Plant material was extracted using seven different protocols (A–G). A: one part of plant material was twice extracted with 10 parts of water at ambient temperature for 30 min in an ultrasonic bath. The extracts were pooled and freeze dried. B: one part of plant material was extracted twice with 10 parts of methanol at ambient temperature for 30 min in an ultrasonic bath. The extracts were pooled and dried using a rotary evaporator. C: one part of plant material was extracted twice with 10 parts of dichloromethane at ambient temperature for 30 min in an ultrasonic bath. The extracts were pooled and dried using a rotary evaporator. D: one part of plant material was twice extracted with 10 parts of methanol at ambient temperature for 30 min in an ultrasonic bath. The extracts were pooled, mixed with the same volume of water and kept in the fridge for 12 h. The extract was filtered, the methanol removed using a rotary evaporator and then freeze dried. E: one part of plant material was extracted twice with 10 parts of dichloromethane at ambient temperature for 30 min in an ultrasonic bath. The residual plant material was air dried and then extracted twice with 10 parts of 50% methanol at ambient temperature for 30 min in an ultrasonic bath. The 50% methanol extracts were pooled, the methanol removed using a rotary evaporator and then freeze dried. F: one part of plant material was extracted twice with 10 parts of dichloromethane at ambient temperature for 30 min in an ultrasonic bath. The residual plant material was air dried and then extracted twice with 10 parts of methanol at ambient temperature for 30 min in an ultrasonic bath. The methanol extracts were pooled, mixed with the same volume of water and kept in the fridge for 12 h. The extract was filtered, the methanol removed using a rotary evaporator and then freeze dried. G: one part of plant material was twice extracted with 10 parts of methanol at ambient temperature for 30 min in an ultrasonic bath. The extracts were pooled, mixed with the same volume of water and kept in the fridge for 12 h. The extract was filtered and subjected to rotary evaporation; water was added during the evaporation until the distillate became colorless. This procedure G removes juglone and other steam-volatile compounds from the extract.

### 3.4. LC-MS Measurements

For the preparation of LC-MS samples, each extract was dissolved in methanol to a concentration of 20 mg/mL by 5-min treatment in an ultrasonic bath. The solutions were centrifuged for 5 min at 14,680 rpm and the supernatant subjected to LC-MS analysis. Five random samples were injected before valid data was recorded in order to saturate the column with sample matrix. Each extract was then measured twice in one sequence without interruption. The order of the samples was changed randomly after the first measurement. Separation was performed on an Ultimate 3000 RS Liquid Chromatography System (Dionex Softron GmbH, Germering, Germany) over a Dionex Acclaim RSLC 120, C18 column (2.1 mm × 100 mm, 2.2 μm) with a binary gradient (A: water with 0.1% formic acic; B: acetonitrile with 0.1% formic acid) at 0.4 mL/min. 0 to 5 min: Isocratic at 5% B; 5 to 37 min: Linear from 5% to 100% B; 37 to 47 min: Isocratic at 100% B; 47 to 48 min: Linear from 100% to 5% B; 48 to 55 min: Isocratic at 5% B. The injection volume was 2 μL. Eluted compounds were detected using a Dionex Ultimate DAD-3000 RS over a wavelength range of 200–400 nm and a micrOTOF-QII time-of-flight mass spectrometer (Bruker Daltonics, Bremen, Germany) equipped with an Apollo electrospray ionization source in positive mode at 2 Hz over a mass range of *m*/*z* 50–1000 using the following instrument settings: Nebulizer gas nitrogen, 4 bar; dry gas nitrogen, 9 L/min, 200 °C; capillary voltage −4500 V; end plate offset −500 V; transfer time 100 μs, prepulse storage 6 μs; collision energy and collision RF settings were combined to each single spectrum of 2500 summations as follows: 1250 summations with 80 eV collision energy and 130 Vpp + 625 summations with 16 eV collision energy and 130 Vpp + 625 summations with 16 eV collision energy and 130 Vpp. MS/MS scans were triggered by AutoMS2 settings within a range of *m*/*z* 150–1000 (toggled off for fingerprints to be used in PLS models). Internal dataset calibration (HPC mode) was performed for each analysis using the mass spectrum of a 10 mM solution of sodium formiate in 50% isopropanol that was infused during LC re-equilibration using a divert valve equipped with a 20 μL sample loop.

### 3.5. Antiprotozoal Assays

Antiprotozoal testing of all extracts was performed at the Swiss TPH in Basel, Switzerland. For activity against TB, the strain STIB 900 (bloodstream trypomastigotes) was tested. For TC, the strain Tulahuen C2C4 (intracellular amastigotes in L6 cells) was used. For LD, activity against the strain MHOM/ET/67/L82 (axenic amastigotes) was determined. For control measurements of cytotoxicity, L6-cells (rat skeletal myoblasts, also used as host cells in the TC assay) were used. All extracts were tested for percent growth inhibition at two concentrations, namely, 2 and 10 μg/mL. IC_50_ values were determined for pure compounds as previously published. For more detailed protocols, see [20].

### 3.6. Calculation of PLS Models 

Before calculating the PLS model, the LC-MS data of all extracts were converted to a bucket table using the Software ProfileAnalysis (Version 2.0, Bruker Daltonik GmbH) using the Find molecular features function for Data Selection with s/n threshold 3, Correlation coefficient 0.6, Minimum compound length 4 spectra, smoothing width 2 and additional smoothing activated. Buckets were generated from 1 to 30 min and from 100 to 1000 *m*/*z*. Advanced bucketing was applied with a retention time tolerance of 0.3 min and an *m*/*z* tolerance of 100 mDa. Data were not normalized within each analysis. Buckets with a value count of less than 50% were ruled out. Afterwards, the bucket table was transferred from ProfileAnalysis to the Software The Unscrambler (Version 9.2, Camo Process AS): The bucket table was exported from ProfileAnalysis in Simca-P + format which was imported from MS-Excel where data were transposed, assorted and exported as CSV. The CSV data were in turn imported by The Unscrambler as ASCII. With The Unscrambler, all buckets were scaled to unit variance (1.00/SDev) and the PLS model was calculated with full cross validation, using the samples’ bucket tables as independent x-data and their bioactivity (expressed as percent of inhibition at 10 μg/mL) as dependent y-data. To refine the initial model, 3 of the extracts leading to large residues were left out of the model (No. 19, 34 and 36). As a final step, the model was recalculated with the significant x-variables (buckets) selected by Martens’ Uncertainty Test.

### 3.7. Isolation and Structure Elucidation of (**1**)

Green, freeze dried leaves of JR (396 g, collected in 09/2012 in the garden of the IPBP, University of Münster) were macerated with methanol at ambient temperature to yield 80 g of dry extract after evaporation under reduced pressure. A portion (50 g) of this extract was separated over a glass column (60 cm × 5.2 cm, V = 1274 mL) filled with 500 g of Sephadex LH20 as stationary phase. The column was eluted with 3 L of methanol at a flow rate of 1 mL/min. Eluate was collected in portions of 10 mL. The fraction eluting between 1580 and 1650 mL yielded 3 g. 2.5 g of this fraction were separated further on a glass column (60 cm × 4 cm, V = 754 mL) packed with 250 g of silica gel 60 using a step gradient of 1 L ethyl acetate: methanol:water (9:1:1), 0.5 L of ethyl acetate:methanol:water (5:5:1) and 0.5 L of methanol at a flow rate of 1 mL/min. The eluate was collected in portions of 10 mL. The fraction eluting between 430–1480 mL yielded 1.49 g. 50 mg of this fraction were dissolved in 5 mL of methanol and subjected to preparative HPLC. Separation was performed on an HPLC system consisting of two Waters 515 pumps (Waters GmbH, Eschborn, Germany) connected to Waters Pump Control Module II, and a K-2500/A 4080 UV-detector (Knauer Wissenschaftliche Geräte GmbH, Berlin, Germany) over a Eurosphere 100 C18 column, 7 μm, 250 mm × 20 mm (VDS optilab Chromatographietechnik GmbH, Montabaur, Germany) with a binary gradient (A: water, B: methanol) at 10 mL/min. 0 to 15 min: Linear from 30% to 50% B; 15 to 20 min: Isocratic at 50% B; 20 to 23 min: Linear from 50% to 100% B; 23 to 26 min: Isocratic at 100% B; 26 to 41 min: Isocratic at 30% B. UV detection at 304 nm. Pure (**1**) eluted between 10.1 and 13.3 min (10.8 mg).

Isolated **1** was identified by its UV- and high resolution mass spectrum as achieved by LC-MS and NMR experiments (^1^H-NMR, ^13^C-NMR, COSY, HSQC and HMBC) run on a DD2 600 MHz NMR instrument (Agilent Technologies Germany, Waldbronn, Germany). The spectra were referenced to the signal of residual methanol at 3.310 (^1^H) and at 49.00 (^13^C) ppm.

*4-O-β-d-Glucopyranosyl-1,4,5-trihydroxynaphthalene* (*Hydrojuglone glucoside*, **1**): Amorphous white powder; UV (water-acetonitrile with 0.1% formic acid) λ_max_ 223, 306, 324, 339 nm; ^1^H-NMR (D_2_O, 600 MHz) δ 7.68 (1 H, dd, *J* = 8.4, 1.1 Hz, H-8), 7.29 (1 H, dd, *J* = 8.4, 7.6 Hz, H-7), 7.25 (1 H, d, *J* = 8.4 Hz, H-3), 6.83 (1 H, dd, *J* = 7.6, 1.1 Hz, H-6), 6.71 (1 H, d, *J* = 8.4 Hz, H-2), 5.00 (1 H, d, *J* = 7.6 Hz, H-1ʹ), 3.97 (1 H, dd, 12.0; 1.9 Hz, H-6ʹa), 3.77 (1 H, dd, *J* = 12.1, 5.4 Hz, H-6ʹb), 3.56–3.48 (4 H, m, H-2ʹ, H-3ʹ, H-4ʹ, H-5ʹ); ^13^C-NMR (D_2_O, 150 MHz) δ 154.74 (C, C-5), 150.53 (C, C-1), 148.63 (C, C-4), 128.86 (C, C-9), 127.07 (CH, C-7), 117.83 (C, C-10), 114.62 (CH, C-8), 112.94 (CH, C-2), 112.07 (CH, C-6), 108.21 (CH, C-3), 105.63 (CH, C-1ʹ), 78.72 (CH, C-5ʹ), 78.18 (CH, C-3ʹ), 75.27 (CH, C-2ʹ), 71.42 (CH, C-4ʹ), 62.59 (CH_2_, C-6ʹ); HRESIMS *m/z* 339.1090 [M + H]^+^ (calcd for C_16_H_19_O_8_, 339.1074). HRESIMS/MS of [M + H]^+^ *m/z* 339.1058 (15), 321.1078 (3), 303.0876 (11), 285.0753 (6), 273.0863 (4), 255.0575 (3), 243.0726 (6), 201.0579 (3), 177.0565 (100), 159.0465 (3), 131.0504 (7), 107.0531 (6).

## 4. Conclusions

In our PLS model, compound **1** was predicted to account for the observed activity against TB. Antitrypanosomal activity of **1** was confirmed with the isolated compound. Consequently, the reflection on PLS models with chemical fingerprints as independent and bioactivity data as dependent variables has proven suitable for the identification of bioactive compounds from complex extracts, thereby recommending itself as an alternative to bioassay-guided isolation.

The PLS approach outclasses bio-guided isolation in that biological testing does not have to be iterated in between successive isolation procedures, but analytically guided targeted isolation can be applied once an active has been predicted by PLS. This was of particular advantage in this study, as the biotesting was performed in the laboratory of one cooperation partner remote from the chemical laboratories of the other, where LC-MS measurements and isolation took place. Thus, the prediction of actives from multiple variable samples by PLS or comparable methods of multivariate statistics represents an ideal platform for international cooperation. The required sample variability was successfully achieved by combining different plant materials from *Juglans* spp. with different sample preparation techniques. Though senescent material has not proven to contain NQ with improved bioactivity as initially hypothesized, its inclusion has aided the successful bioactivity prediction of **1**, as many of the extracts less active against TB derived from senescent source materials and contained lower amounts of **1**.

As cytotoxicity is a limiting factor for the application of many NQ, a particular interesting property of **1** is its superior selectivity index towards TB that outclasses established antiprotozoal NQs like lapachol. The much lower IC_50_ values of the benzoquinone glucoside arbutin suggests that **1** is not acting via unspecific toxic effects attributable to its phenolic character while differences in redox potential might play a role. Compared to juglone, the hydroquinone glucoside **1** shows an increased selectivity index. As TB is known to hold β-glycosidase activity [21], it seems reasonable that **1** enters the parasite cells as a prodrug to finally yield the NQ juglone, which is almost four times more active against TB and still displays an SI value > 10. This may explain the improved selectivity index in the cell culture models, even though the exact fate of **1** in trypanosomes and mammalian cells remains to be elucidated.

To our knowledge, this is the first description of a glucosylated NQ-related compound showing activity against protozoans. Especially the low cytotoxicity of the glycosylated compound leading to higher selectivity against the protozoans compared to simple NQ is of interest and suggests screening further glycosidic NQ-related compounds for antiprotozoal activity.
